# Structural and Functional Changes in the Coupling of Fascial Tissue, Skeletal Muscle, and Nerves During Aging

**DOI:** 10.3389/fphys.2020.00592

**Published:** 2020-06-24

**Authors:** Alberto Zullo, Johannes Fleckenstein, Robert Schleip, Kerstin Hoppe, Scott Wearing, Werner Klingler

**Affiliations:** ^1^ Department of Sciences and Technologies, University of Sannio, Benevento, Italy; ^2^ CEINGE Advanced Biotechnologies, Naples, Italy; ^3^ Department of Sports Medicine, Institute of Sports Sciences, Goethe-University Frankfurt, Frankfurt, Germany; ^4^ Department of Sport and Health Sciences, Technical University Munich, Munich, Germany; ^5^ Department of Sports Medicine and Health Promotion, Friedrich-Schiller University Jena, Jena, Germany; ^6^ Department of Anaesthesiology, Würzburg University, Würzburg, Germany; ^7^ Faculty of Health School, Queensland University of Technology, Brisbane, QLD, Australia; ^8^ Fascia Research Group, Department of Experimental Anaesthesiology, Ulm University, Ulm, Germany; ^9^ Department of Anaesthesiology, SRH Hospital Sigmaringen, Sigmaringen, Germany

**Keywords:** aging, connective tissue, fascia, skeletal muscle, nerve

## Abstract

Aging is a one-way process associated with profound structural and functional changes in the organism. Indeed, the neuromuscular system undergoes a wide remodeling, which involves muscles, fascia, and the central and peripheral nervous systems. As a result, intrinsic features of tissues, as well as their functional and structural coupling, are affected and a decline in overall physical performance occurs. Evidence from the scientific literature demonstrates that senescence is associated with increased stiffness and reduced elasticity of fascia, as well as loss of skeletal muscle mass, strength, and regenerative potential. The interaction between muscular and fascial structures is also weakened. As for the nervous system, aging leads to motor cortex atrophy, reduced motor cortical excitability, and plasticity, thus leading to accumulation of denervated muscle fibers. As a result, the magnitude of force generated by the neuromuscular apparatus, its transmission along the myofascial chain, joint mobility, and movement coordination are impaired. In this review, we summarize the evidence about the deleterious effect of aging on skeletal muscle, fascial tissue, and the nervous system. In particular, we address the structural and functional changes occurring within and between these tissues and discuss the effect of inflammation in aging. From the clinical perspective, this article outlines promising approaches for analyzing the composition and the viscoelastic properties of skeletal muscle, such as ultrasonography and elastography, which could be applied for a better understanding of musculoskeletal modifications occurring with aging. Moreover, we describe the use of tissue manipulation techniques, such as massage, traction, mobilization as well as acupuncture, dry needling, and nerve block, to enhance fascial repair.

## Introduction

In animal species, aging is associated with substantial modifications to the molecular determinants of cells, which alter their morphology, activity, and functional properties. These events affect the characteristics of tissues and organs, thus resulting in overall decayed performance of the organism (Narici et al., 2008; Zullo et al., 2017). Indeed, during aging, human skeletal muscle, connective tissue, and the nervous system undergo consistent modifications, which can impair the ability to perform daily activities and, as a consequence, their health-related quality of life (Netuveli et al., 2006; Reid and Fielding, 2012; Diehr et al., 2013). To promote healthy aging and reduce the burden of aging, it would be important to define the time-dependent alterations impairing the neuromuscular system. Also, sensitive, specific, and non-invasive methods to investigate the structural and morphological changes occurring in these tissues during aging are needed, and therapeutic interventions should be implemented. In this review, we outline the structural and functional changes occurring within skeletal muscle, fascial tissue, and nervous system during aging. Moreover, we summarize the evidence on the age-dependent impairment of the coupling between these tissues and the effect of inflammation in aging. Finally, we describe some approaches for the study and the treatment of age-associated modifications in the neuromuscular system, such as imaging and ultrasound-based methods, as well as tissue manipulation techniques.

## Skeletal Muscle and Aging

Sarcopenia, a gradual loss in muscle mass occurring in humans during aging, is detectable in the third decade of life and progressively increases with age (Janssen et al., 2000; Narici and Maffulli, 2010). It has been estimated that, during aging, there is a 30–50% reduction in the number and 10–40% decrease in the size of skeletal muscle fibers, which is associated with a decay of muscle performance (Lexell et al., 1988; Lexell, 1995; Janssen et al., 2002; Doherty, 2003). In particular, a reduction in skeletal muscle mass has been estimated to be 0.37 and 0.47% per year in women and men, respectively (Mitchell et al., 2012). Clinical studies in old adults have shown that muscle strength declines at a greater rate than muscle mass (Frontera et al., 2000; Goodpaster et al., 2006; Delmonico et al., 2009). Therefore, factors other than muscle mass must also contribute to the decay of muscle performance in aging.

In humans, three isoforms (type I, IIa, and IIx) of the myosin heavy chain (MHC) are present, with different combinations and proportions in individual muscles (Schiaffino and Reggiani, 1994, 1996). Each isoform has specific molecular features, which in turn, determine muscle characteristics (Trappe, 2009). There is evidence that the composition of the contractile apparatus of muscle, i.e., the isoforms of the MHC, varies with aging (Trappe, 2009). In the vastus lateralis of old adults, the percentage of muscle fibers expressing both MHC types I (MHC-I) and IIa (MHC-IIa) and MHC-IIa and IIx (MHC-IIx) is higher, and the percentage of muscle fibers expressing only MHC-I and MHC-IIa is lower than those of young adults (Klitgaard et al., 1990). Moreover, in the sternocleidomastoid muscle (SCM) of old, compared to young, adults, the proportion of slow-twitch fibers is higher, the share of hybrid MHC-IIa/MHC-IIx fibers is reduced, the proportion of fast-twitch fibers with MHC-IIa and IIx is lower, and the proportion of fibers with the isoform MHC-IIx is reduced (Meznaric et al., 2018). This leads to a shift toward a slower phenotype of muscle fibers and suggests that aging induces the replacement of fast-twitch with slow-twitch motor units (Meznaric et al., 2018). Interestingly, the age-dependent decrease in the number and size of muscle fibers involves mainly type II, but not type I muscle fibers (Larsson, 1978; Lexell et al., 1988; Lexell, 1995; Martel et al., 2006; Snijders et al., 2009).

As for fiber structure, it has been demonstrated that pennation angles in the vastus lateralis muscle diminish with advancing age in women (Kubo et al., 2003). Such architectural modification results in a reduced number of parallel fibers and negatively impacts on muscle force generation (Thom et al., 2007). Moreover, besides changes in structural proteins, alteration of contractile and metabolic proteins, as well as calcium-handling proteins of skeletal muscle cells also take place in the elderly (Godard et al., 2002; Thompson, 2002). The impairment of excitation-contraction coupling, the increase of intermuscular adipose tissue, the alteration of intramuscular adipose tissue, and the amount of extracellular water relative to muscle volume with aging could also promote the decline in muscle performance (Rice et al., 1989; Yamada et al., 2010; Marcus et al., 2012; Beavers et al., 2013; Hausman et al., 2014; Tieland et al., 2018).

As for muscle function, data from studies in animal models and humans demonstrated that the strength and the shortening velocity of muscle fibers decrease with advancing age (Lauretani et al., 1985; Frontera et al., 2000; Thompson, 2002; D’Antona et al., 2003; Miljkovic et al., 2015; Del Campo et al., 2018). A study in a large cohort of adults demonstrated a significant reduction in isometric, concentric, and eccentric peak torque in the extensor muscles of the knee in old, compared to young, adults (Lindle et al., 1997). Also, a 16.6–40.9% reduction in muscular strength has been estimated by comparing young adults (<40 years) with old one (>40 years) (Keller and Engelhardt, 2014). A research employing magnetic resonance imaging (MRI) has demonstrated that maximum voluntary contraction, when normalized to the anatomical cross-sectional area of muscle, is lower in the elderly than healthy young adults (Morse et al., 2005). Although several pieces of evidence have been accumulated on the functional decline of skeletal muscle during aging, this issue is still disputed (Lexell et al., 1988; Trappe et al., 2003; Short et al., 2005a; Canepari et al., 2010). Indeed, some studies report that the power of human muscle fibers, normalized for cell size, does not change with age (Trappe et al., 2003; Malisoux et al., 2007; Raue et al., 2009).

The wide remodeling of skeletal muscle occurring with age also influences its mechanical properties. Kocur and colleagues demonstrated that stiffness and tone of the upper trapezius (UT) and SCM increase with age, and elasticity decreases (Kocur et al., 2017). On the contrary, the viscoelastic features of these muscles during movement are not significantly affected (Kocur et al., 2017). The analysis of muscle tone and mechanical properties of rectus femoris and biceps brachii muscles in old and young adults revealed that aging is associated with an increase of muscle tone, and stiffness, and a decrease in elasticity (Agyapong-Badu et al., 2016). Also, the sternocleidomasteoid and upper trapezius muscles undergo a decrease in elasticity and an increase in stiffness in women in a seated position (Kocur et al., 2019). These parameters varied about 1.5% per year, and, among all the possible etiologic factors analyzed (BMI, age, and head posture), only aging was found to be a major correlate (Kocur et al., 2019). Neuromuscular performance is also impaired with aging, as demonstrated in a study that evaluated both the time needed to perform specific muscular activities and muscular functional features (Haus et al., 2007b). The authors showed that the time to climb stairs, rise from a chair, and walk a set distance significantly increased and the quadriceps muscle volume, strength, and power significantly decreased in aged adults (Haus et al., 2007b). These alterations did not match changes in intramuscular endomysial collagen nor in enzymatically-mediated collagen cross-linking, which remained constant, but matched a 200% increase in the advanced glycation end-product (AGE), pentosidine (Haus et al., 2007b). The accumulation of AGEs in the intramuscular connective tissue, probably due to age-dependent decrease in collagen turnover, is associated with the formation of non-enzymatically-mediated collagen cross-linking (Verzijl et al., 2000; Haus et al., 2007b). This observation led the authors to hypothesize that the accumulation of AGEs in the intramuscular connective tissue of old adults might be responsible for the increase in muscle tissue stiffness and decrease in passive viscoelastic properties, thus impairing muscle function.

Skeletal muscle is highly responsive to activity, i.e., it remodels and adapts its metabolism and structure to meet the body’s needs (Thompson, 2002; Potthoff et al., 2007). Aging affects the architecture and the function of the skeletal muscle, but there are conflicting reports about the level of muscular adaptive capacity in the elderly (Rogers and Evans, 1993; Kim and Thompson, 2013; Gheller et al., 2016). However, based on some pieces of evidence, aged skeletal muscle still maintains a certain degree of plasticity; therefore, it can undergo structural and functional changes in response to physiological stimuli (Joanisse et al., 2017). In particular, exercise training has been demonstrated to stimulate skeletal muscle fiber hypertrophy and increase mitochondrial oxidative capacity in aged humans and animal models, resulting in increased muscle mass and strength (Frontera et al., 1988; Joseph et al., 2016; White et al., 2016; Joanisse et al., 2017; Kim et al., 2017). At the same time, inactivity induces a decrease in skeletal muscle mass, and strength independently of age, but the recovery after immobility is impaired in aged humans and animal models (Zhang et al., 2018a,b; Oikawa et al., 2019).

At the molecular level, reactive oxygen species (ROS) and PGC-1alpha signaling, as well as the autophagic machinery, have been suggested as important determinants contributing to metabolic adaption and remodeling of skeletal muscle after physical activity (Ferraro et al., 2014). Skeletal muscle fibers during their activity produce ROS, mainly due to the activity of mitochondria, NADPH oxidase, xanthine oxidase, and phospholipase A2 (Szentesi et al., 2019). In turn, ROS activate several signaling pathways, which are pivotal for different cellular processes, such as proliferation, protein synthesis and degradation, ROS detoxification, and muscle fiber contraction (McDonagh, 2016). ROS, at physiological levels, have a crucial role in the activation of adaptive responses to exercise in skeletal muscle cells (Ji, 2015). Indeed, mitochondrial biogenesis, antioxidant activity, inflammation, protein turnover, apoptosis, and autophagy are upregulated. The principal molecular elements of these signaling cascades are NF-kB, MAPK, and PGC-1alpha. When the levels of ROS greatly exceed the cellular antioxidant capacity, deleterious oxidative modifications occur to the structures and the molecules of the skeletal muscle fibers, thus impairing their function. Aging is associated with increased levels of oxidative stress in the skeletal muscle tissue, which is the accumulation of reactive oxygen and nitrogen species. As a result, the skeletal muscle undergoes structural and functional modifications, which reduce muscle mass, strength, and function (Ji, 2015). Indeed, satellite cells become dysfunctional, the rate of protein breakdown prevails over that of protein synthesis, muscle fibers activate apoptotic processes, intracellular calcium homeostasis is altered, and excitation-contraction coupling is impaired (Szentesi et al., 2019). Studies in humans and mice showed that mitochondria of aged skeletal muscles have impaired mitochondrial NADH redox potential and reduced oxidative capacity (Conley et al., 2000; Claflin et al., 2015; Nelson et al., 2019). As a consequence, aged muscles accumulate oxidized proteins, and mitochondrial dysfunction, which, in turn, lead to increased ROS production and oxidative damage in the cells (Short et al., 2005b; Cerullo et al., 2012; Bratic and Larsson, 2013; Gomes et al., 2017). Besides, other cellular antioxidant enzymes, such as catalase, glutathione transferase, and superoxide dismutase, are also significantly reduced in skeletal muscle fibers of old adults, and exercise-activated redox signaling pathways are weakened (Cerullo et al., 2012; Ji, 2015). A proteomic study in mice demonstrated that aging is correlated with reduced levels of redox-sensitive proteins involved in the energy metabolism of skeletal muscle fibers, thus affecting the cellular responses to oxidants (McDonagh et al., 2014). These data were also supported by a recent study in humans, which showed that sarcopenia is associated with altered expression of genes regulating mitochondrial energy production in skeletal muscle (Migliavacca et al., 2019). Indeed, the PGC-1alpha/ERRalpha signaling, oxidative phosphorylation, and proteostasis are downregulated. As a result, mitochondria are dysfunctional and their number is reduced, thus further exposing skeletal muscle fibers to oxidative damage (Migliavacca et al., 2019).

Experimental studies indicate a negative effect of aging also on skeletal muscle plasticity, which is the potential of this tissue to modify its structural and functional features in response to environmental changes (Verdijk et al., 2007; Jee and Kim, 2017; Suetta, 2017). The regrowth response to physical inactivity-induced atrophy is also hindered in aged muscles (Pisot et al., 2016).

As for the regenerative potential of skeletal muscle during aging, it has been reported that satellite cells, the quiescent adult stem cells of skeletal muscle, gradually lose their potential to regenerate skeletal muscle with advancing age (Yin et al., 2013; Sousa-Victor et al., 2014; Joanisse et al., 2016). Indeed, experimental evidence from studies in both humans and animal models suggests that aging is associated with a reduction in satellite cell self-renewal and myogenic competences, and possibly a decrease in their number, thus resulting in an impaired regeneration of skeletal muscle tissue (Corbu et al., 2010; Sousa-Victor et al., 2014; Brack and Muñoz-Cánoves, 2016; Joanisse et al., 2016). At the molecular level, aging drives a dysregulation of important molecular signaling pathways, such as FGF2/Sprouty1, Notch, p38 MAPK, JAK-STAT3, and p16(INK4a) in satellite cells, and impairs the microenvironment feeding and regulating the muscle stem cell niche (Sousa-Victor et al., 2014; Parker, 2015; Snijders et al., 2015; Brack and Muñoz-Cánoves, 2016; Joanisse and Parise, 2016; Stearns-Reider et al., 2017; Etienne et al., 2020; Levi et al., 2020).

To counteract the impairment of skeletal muscle with aging, three main approaches have been pursued: physical activity, antioxidant dietary supplementation, and regenerative medicine therapies. Some pieces of evidence showed that regular exercise can reduce the detrimental effects of aging on skeletal muscle. In particular, it has been reported, both in humans and animal models, that regular physical activity can attenuate the age-dependent decrease in the number of mitochondria and proteins involved in the excitation-contraction coupling, thus delaying the impairment in muscle function (Zampieri et al., 2015; Csernoch et al., 2017). Physical inactivity also damages the structure and function of skeletal muscle fibers. Moreover, in old adults, besides a weakened ROS scavenging system, dietary intake of antioxidants is often reduced (Damiano et al., 2019). In this context, data from studies in humans and animal models indicate that muscular activity and/or dietary supplementation with antioxidant compounds, such as l-ascorbic acid, tocopherols, carotenoids, flavonoids, or polyphenols, exert some beneficial effects on age-related skeletal muscle decline (Cesari et al., 2004; Pierno et al., 2014; Anton et al., 2015; Cartee et al., 2016; Jee and Kim 2017; Muhammad and Allam, 2018; Damiano et al., 2019). However, it should be taken into account that the effects of physical exercise and/or dietary supplementation with antioxidants on skeletal muscle aging are still debated due to discordant results (Marcell, 2003; Ji, 2015; Cartee et al., 2016; Flack et al., 2016; White et al., 2016; Distefano and Goodpaster, 2018; Damiano et al., 2019; Szentesi et al., 2019).

In the last decades, thanks to the progress of regenerative medicine, new approaches for improving skeletal muscle regeneration in humans have been developed. Indeed, cell therapies based on the delivery of myogenic stem cells have been largely used also in preclinical studies. Several sources of myogenic cells have been employed, so far: satellite cells, muscle-derived stem cells, myoblasts, mesoangioblasts, hTERT-immortalized muscle precursor cells, pericytes, CD133^+^ cells, hematopoietic stem cells, mesenchymal stem cells, perivascular stem cells, interstitial cells, pluripotent stem cells isolated from the dental pulp, embryonic stem cells, and induced pluripotent stem cells (iPSCs) (Hall et al., 2017; Del Carmen Ortuño-Costela et al., 2019; Marg et al., 2019; Mueller and Bloch, 2019). As a result, the delivery of these myogenic cells led to some improvements in muscle function and regeneration in mammalian animal models (Rao et al., 2018; Mueller and Bloch, 2019). To enhance the effectiveness of these regenerative therapies, bioactive molecules, such as TGF-β, IGF-I, fibrin, keratine, as well as ECM-based bioscaffolds, have been combined with myogenic cells (Brown et al., 2012; Dziki et al., 2016; Fuoco et al., 2016; Jiao et al., 2018; Pollot et al., 2018; Mueller and Bloch, 2019). However, we are still far from effective treatments to use in clinical practice for skeletal muscle regeneration due to the difficulties associated with cell preparation, the limited engraftment of myogenic cells, the incomplete differentiation of myogenic cells *in situ*, the partial integration with host cells, and tumorigenic risk of immortalized cells. In the context of pharmacological intervention, many different drugs have been tested for improving muscle regeneration. Indeed, anti-inflammatory drugs have been used for blocking the anti-regenerative effect of chronic inflammation, hormones, and growth factors for stimulating cell growth; urocortin II for stimulating muscle fiber growth pathways; and myostatin for activating satellite cells. Indeed, urocortin II and myostatin showed an interesting potential for improving muscle hypertrophy and hindering the age-dependent loss of muscle tissue in animal models (Hinkle et al., 2003; Rodriguez et al., 2014; Cohen et al., 2015; Naranjo et al., 2017; Saul et al., 2019).

## Connective Tissue and Aging

Besides its influence on skeletal muscle, aging also results in modification of cells and extracellular matrix of myofascia and tendons (Barros et al., 2002; Haus et al., 2007a; Trappe, 2009; Kragstrup et al., 2011). Muscular fascia is composed of many different molecules, such as structural proteins (collagens, laminins, fibronectin, vitronectin, tenascin, and elastin), growth factors (TGFs and IGFs) glycosaminoglycans, proteoglycans, metalloproteinases, cytokines, and water (Eyre et al., 1984; Reiser et al., 1992; McCormick, 1999). Fascia, due to its structure and composition, has elastic, viscoelastic and plastic properties that strongly influence the biomechanical features of the locomotory apparatus, as demonstrated both in humans and animal models (Kovanen et al., 1988; Goldspink et al., 1994; Kjaer, 2004; Avila Gonzalez et al., 2018; Blottner et al., 2019; Schleip et al., 2019). Muscular connective tissue changes with advancing age, as reported also in studies in animal models. In particular, its thickness and the amount of collagen cross-linking increase, and its elasticity decreases (Alnaqeeb et al., 1984; Gosselin et al., 1998; Ducomps et al., 2003; Wilke et al., 2019; Etienne et al., 2020). Additionally, the composition of the muscular connective tissue varies; as an example, the amount of collagen type IV rises, and that of type VI reduces (Etienne et al., 2020; Levi et al., 2020). As a result, the extracellular matrix becomes more rigid and muscles increase their stiffness, thus resulting in impaired muscle function (Alnaqeeb et al., 1984; Kovanen et al., 1984; Gosselin et al., 1998; Willems et al., 2001; Ducomps et al., 2003; Etienne et al., 2020).

Studies in humans and animal models showed that with advancing age the basal lamina becomes thicker and destructured, and the content of collagen type IV, laminin, and the antimyogenic cytokine, osteopontin, increases in skeletal muscle, thus hindering its regenerative potential (Kovanen et al., 1988; Grzelkowska-Kowalczyk, 2016). Moreover, a decrease in the number of fibroblasts and stem cells in tendon during aging has been also reported in humans and animal models (Squier and Magnes, 1983; Nakagawa et al., 1994; Zhou et al., 2010; Ruzzini et al., 2013).

A recent study by Wilke and collaborators demonstrated that aging is associated with variation in the thickness of the fascia. Indeed, the age-related modifications are specific for different body sites. Fascial thickness of the lower limb decreases with age (−12.3–25.8%), whereas fascia of the low back region increases (+40.0–76.7%) (Wilke et al., 2019). These changes in connective tissue have been suggested to reduce joint flexibility (Wilke et al., 2019). Moreover, the amount of intramuscular connective tissue in the gastrocnemius muscle of elderly adults is reportedly higher than that of young adults (Csapo et al., 2014). Although connective tissue content may change with aging, the amount of cross-linking of intramuscular collagen remains stable, according to the results of a study in the vastus lateralis of young and old adults (Haus et al., 2007a). Aging results also in a reduction in water content within tendon, as reported in the Achilles tendon of old rabbits (Ippolito et al., 1980), an increase in cross-links between tendon fibrils and a decrease in collagen content and density (Vogel, 1978; Haut et al., 1992; Couppe et al., 2009; Svensson et al., 2015). Reducing sugars can link to amino acids of collagen fibers and generate AGEs, which accumulate throughout life and result in increased tissue stiffness and strength (Bank et al., 1999; DeGroot et al., 2001; Suzuki et al., 2008).

In addition, reduced elasticity and increased stiffness of aged musculoskeletal system can also be caused by degeneration of connective tissue, which leads to reduced joint mobility in the elderly (Kocur et al., 2019). At the molecular level, it has been reported that Wnt signaling, the expression of metalloproteinase (MMP) and tissue inhibitor of metalloproteinase genes, which regulate ECM degradation and remodeling, change with advancing age (Phillip et al., 2015; Birch, 2018; de Sousa Neto et al., 2018). Barros and colleagues demonstrated that elastic fibers of the cervical interspinous ligaments undergo fragmentation and degeneration, and oxytalan fibers, which confer resistance to the tissue, degrade during aging (Barros et al., 2002). As a consequence, ligaments are more susceptible to ruptures following mechanical stress (Barros et al., 2002).

Despite age-related modifications of connective tissue, the effect of these changes on the mechanical properties of tendons, such as strength, stiffness, and elasticity, is still debated, due to conflicting reports (Svensson et al., 2015). Interestingly, a recent pilot study demonstrated that acute resistance exercise differentially affected young and old adults in the context of metalloproteinase gene expression (Wessner et al., 2019). These results support the evidence of stimulus-dependent ECM remodeling in the elderly.

## Coupling Between Fascia, Skeletal Muscle and Aging

Muscles and fascia cooperate for the correct functioning of the locomotory apparatus. Their intimate relationship makes the performance of movement strictly dependent on the status of both of them. As an example, appropriate preparation of fascial structures by a warm-up and stretching protocols is essential for optimal results and minimal risk of injury in physical exercises (Yahia et al., 1993; Mattieni et al., 2009; Wang et al., 2009). The structural and molecular changes of skeletal muscle and fascia with advancing age influence the transmission of force in the locomotory system (Wilke et al., 2018). Intramuscular fascia connects different muscle fibers and muscle bundles within the muscle to form the force-generating structure connected to the bone (Turrina et al., 2013). Indeed, studies in rats showed that the force generated by muscle fiber contraction is transmitted both longitudinally and laterally, through the intramuscular fascia, to the surrounding muscle fibers, till the tendon and the bones (Huijing and Jaspers, 2005). Moreover, molecular features, structure, and orientation of fascial fibers determine how force is transmitted and transferred through connective tissue to other surrounding elements (Stecco et al., 2013 Wilke et al., 2018). In humans, recent data showed that the length of muscle fascicles is affected by the coupling between muscle and fascia (Pamuk et al., 2016; Karakuzu et al., 2017). Indeed, heterogeneous fascicle strains have been detected in the medial gastrocnemius muscle following submaximal plantar flexion activity or knee extension. This result has been explained by epimuscular force transmission (Pamuk et al., 2016; Karakuzu et al., 2017). Intermuscular mechanical interactions also have important implications in patients affected by cerebral palsy (CP), who suffer from motor disability. It has been suggested that the co-activation of antagonistic muscles of the knee, which causes the limited joint motion in CP patients, is due to altered force transmission through myofascial structures (Kaya et al., 2019, 2020). This makes fascia a key structural and functional element of the contractile apparatus of muscle. As a consequence, the mechanical features of the entire muscle cannot be merely described as the sum of the isolated fascia and muscle fibers.

The fluid component of fascia assists in the hydrostat function of the tissue and dissipates energy, whereas the elastic component, made by fibrous protein structures, can store and release elastic energy (Huijing et al., 2003; Özkaya et al., 2017). Besides the capacity of fascial components to generate force by myofibroblast contraction, fascia can also modulate its composition and structure in response to biomechanical stimulation, thus allowing its adaptation over time to meet the body’s needs (Blottner et al., 2019). Several studies have demonstrated that muscles located in anatomically separate body regions can exchange tensional stress through a tight link and cooperation with fascial structures, thus contributing to the accomplishment of locomotory tasks and movement proprioception (Vleeming et al., 2014; Blottner et al., 2019). In the anatomical compartment comprised between leg and trunk, this complex architecture has been described as a myofascial force transmission chain (Myers, 2013; Wilke et al., 2016).

Data collected so far suggest that myofascial force transmission is extremely relevant during muscle lengthening, such as in eccentric contractions and stretching activity (Yucesoy et al., 2005; Huijing, 2009; Yucesoy, 2010; Wilke et al., 2018). Indeed, the presence of intermuscular mechanical interactions in the lower leg muscles under passive joint motion has been clearly demonstrated by several ultrasound-based studies (Bojsen-Møller et al., 2010; Tian et al., 2012; Le Sant et al., 2017; Ateş et al., 2018).

Some pieces of evidence indicate that the close interaction between muscular and fascial structures and the physical continuity of connective tissue along the myofascial chain weakens with age, thus reducing the magnitude of mechanical force transmission (Wilke et al., 2019). In this context, it has been shown that alteration in myofascial force transmission can influence the effects of nonlocal exercises, as the self-myofascial release of the plantar fascia on hamstring extensibility (Wilke et al., 2019). Fascial tissue can densify and develop fibrosis with age, thus reducing muscular force production and joint range of motion (Pavan et al., 2014; Zhang and Gao, 2014). Moreover, decreased physical mobility occurring in the elderly could be partially explained by increased stiffness and reduced elasticity of the extracellular matrix due to dehydration and increased collagen content (Sölch, 2015). Indeed, during aging, the connective tissue of patellar tendon increases fiber cross-links and reduces collagen content, and Achilles tendon and plantar fascia diminish their connecting fibers (Snow et al., 1995; Couppe et al., 2009).

## Connective Tissue, Inflammation, and Aging

A key feature of aging tissue is, also, the so-called inflammaging, which describes a low-grade chronic systemic inflammation in the absence of overt infection. This “sterile” inflammation is a highly significant risk factor for morbidity and mortality in elderly people. Chronic inflammation influences tissue functions *via* several mechanisms: persistent production of reactive molecules by infiltrating leucocytes might damage structural elements of tissues; production of cytokines might modulate inflammatory responses and alter phenotypes of nearby cells (Franceschi and Campisi, 2014). Most of the inflammatory responses take place in the extracellular matrix, which can interact with immune cells and change their functions, thereby influencing tissue regeneration. Although early inflammation after tissue damage is important for remodeling and adaptations, decreased inflammation seems to be associated with improved tissue regeneration and gains of muscle strength (Zügel et al., 2018).

## Motor System and Aging

As for the effect of aging on the motors system, data showed that atrophy of the motor cortex, modifications of neurotransmission, reduced motor cortical excitability, and plasticity occur in the nervous system. These changes also result in impaired muscle performance (Tieland et al., 2018). Alterations in nervous system structure and function contribute to a decline in skeletal muscle efficiency with age, *via* a reduction in motor coordination and muscle strength. Loss of neural processes impairs the control and activity of skeletal muscle fibers. Indeed, the occurrence of age-related reduction in descending drive and an increase in the threshold of excitability of the corticospinal tract has been reported by studies in humans (Rossini et al., 1992; Clark and Taylor, 2011; Unhjem et al., 2015). As demonstrated in mouse and rat models, aging is also associated with a decline in reinnervation of muscle fibers, accumulation of denervated fibers, and muscle atrophy. This phenomenon is probably caused by a reduced neurotrophil-mediated axonal sprouting following denervation (Aare et al., 2016). Moreover, aging leads to motor unit remodeling, which leads to an alteration in the fiber type composition, i.e., the replacement of type II with type I muscle fibers (Thompson, 2002). Reduction in the number of motor units is associated with the decay of muscle size and function in aged mice (Sheth et al., 2018). Aging also results in impaired movement coordination, due to a decreased control of motor output, as demonstrated in the first dorsal interosseous muscle of elderly subjects during submaximal contractions (Galganski et al., 1993). Old adults, have 40% reduction in the total number of motor units, 50% enlargement of remaining motor units (low- and moderate-threshold motor units), and an increase in fiber density (Piasecki et al., 2016). Also, differences in inter-joint coordination during squat jumps have been found between young and old adults (Argaud et al., 2019). A study investigating neuromuscular coordination demonstrated a loss and reorganization of muscular patterns in old adults (Vernooij et al., 2016).

Neuromuscular junctions (NMJs) are affected by detrimental modifications in their activity and conformation during aging (Li et al., 2018). Studies in animal models showed that the structural integrity of NMJs is affected by aging. The area of the motor nerve terminal and that of post-synaptic folds is reduced and both nerve terminals and the post-synaptic cluster of acetylcholine receptors (AChRs) undergo fragmentation. Moreover, in aged rodent it has been reported an increase in the branching of nerve terminals, and extra-junctional AChRs, and a reduction in myofibre nerve supply (Kreko-Pierce and Eaton, 2018). Interestingly, the degree of age-dependent degeneration of NMJs shows a certain variability between different muscles and even within the same muscle or the motor unit (Taetzsch and Valdez, 2018). The cause of this NMJs degeneration is still a matter of study, but some pieces of evidence indicate that impaired autophagic pathway and agrin signaling might cause NMJ decline (Li et al., 2018). Data from animal models showed that aged NMJs have a reduced number of mitochondria and accumulate morphological abnormal mitochondria and oxidative damage in the presynaptic plaque (Gonzalez-Freire et al., 2014). Also, it has been reported that aging is associated with altered expression of neurotrophin genes in the neuromuscular system. In particular, neurotrophin-3, neurotrophin-4, and brain-derived neurotrophic factors are downregulated and glial cell-derived neurotrophic factor is upregulated in aged rodent muscles (Kreko-Pierce and Eaton, 2018). The effect of aging on neuromuscular junction transmissions is still debated, due to conflicting results (Piasecki et al., 2016; Willadt et al., 2016). The results collected so far demonstrate that age-dependent changes in the nervous system could promote an impairment in skeletal muscle function (Piasecki et al., 2016). However, in the perspective of healthy aging, several studies in mice demonstrated that the deleterious effects of aging on the structure and function of NMJs can regress due to physical exercise (Fahim, 1997; Valdez et al., 2010; Nishimune et al., 2012; Cheng et al., 2013).

## Imaging and Ultrasound Methods in the Elderly

From the clinical perspective, it is crucial to identify all the alterations that occur in the myofascial tissue with aging. Technological advancements allowed the development of high-resolution instruments for the morphological characterization of myofascia tissue and the assessment of factors that influence the “contractile quality” of skeletal muscle. Conventional imaging methods, such as MRI, the current “gold standard” for the analysis of muscle size, computed tomography (CT), and dual-energy X-ray absorptiometry (DXA), provide precise measures of muscle mass but are suboptimal in reflecting the composition of myofascial tissue. For instance, Hounsfield units in CT images (Engel et al., 2018) or MRI signal intensity can be used to evaluate the composition of the skeletal muscle (Carlier et al., 2016). However, it is difficult to obtain these values for many community-dwelling older people because of the need for special equipment and the relatively high measurement burden (time, cost, and radiation exposure) and the lack of standardization of measurements (Lee et al., 2019). Recently, ultrasound attenuation and echo intensity have emerged as potentially useful indicators of muscle composition. Ultrasonography is non-invasive, easily accessible, and relatively inexpensive compared with other imaging systems such as CT. Several groups of researchers have used ultrasound echo intensity as an index of skeletal muscle composition; whereby, increased amounts of intramuscular fat and fibrosis that occur with pathological aging result in increasing echo-reflection and a brighter ultrasound image (Reimers et al., 1993; Pillen et al., 2009; Watanabe et al., 2018). Although such measures have also been related to muscle strength and functional performance in elderly populations in some studies (Fukumoto et al., 2012; Watanabe et al., 2013; Lopez et al., 2017), they provide only indirect measures of the contractile quality of skeletal muscle *in vivo*. Several alternative ultrasound-based methods have recently been developed to quantify the contractile quality of muscle during dynamic contraction. Elastographic methods are increasingly used to assess the viscoelastic properties of muscle in aging but, as with echo-based measures, their widespread implementation in clinical and research settings is currently limited by low reproducibility (Zaidman et al., 2012; Alfuraih et al., 2018). Although other emerging ultrasound-based approaches including speckle-tracking (Frich et al., 2019), tissue Doppler imaging (Eranki et al., 2013), and axial transmission techniques (Wearing et al., 2016) arguably provide more direct measures of the contractile quality of the muscle-tendon unit *in vivo*, these techniques also suffer from limitations, including susceptibility to decorrelation (speckle tracking), user intervention (feature-based tracking), and dependence on insonation angle (tissue Doppler) (Sikdar et al., 2014). Nonetheless, ultrasound-based measures appear to be promising approaches for quantifying contractile properties of myofascial tissues and have the potential to enhance our understanding of musculoskeletal function with aging and pathology.

## Manipulation Techniques and Fascia Repair

Over the course of a lifetime, fascia can be injured due to excessive or prolonged loading, traumatic events, strenuous physical activity, and surgical procedures. As a consequence of damage, repair mechanisms are activated to restore the original structural and functional features of the tissue (Zullo et al., 2017). Impairment of this process can cause a reduction in the performance of the locomotory apparatus and musculoskeletal disorders (Zuegel et al., 2018). Therefore, strategies improving myofascial regeneration are pivotal. A broad range of tissue manipulation techniques has been proposed to enhance fascial repair. A study in the treatment of tension-type headache suggests a combination of soft tissue techniques and neural mobilization to be most promising in relieving myofascial-induced pain and dysfunction (Ferragut-Garcias et al., 2017). The authors highlight the importance of the treatment stimulus to mechanically stimulate nerve and fascia. Indeed, a study performed on people suffering from delayed-onset muscle soreness showed that the sensitivity of high-threshold mechanosensitive receptors is a predictor of pain and motor impairment (Fleckenstein et al., 2017). Thus, approaches modulating the activity of these receptors may be helpful for functional recovery. This is in line with recent clinical data showing that hands-on based conservative treatments can be effective in relieving pain in injured athletes (Fleckenstein and Banzer, 2019). These treatments include nerve block, injection, ultrasound and laser therapies, manipulation, mobilization, massage, and traction, as well as acupuncture and dry needling. However, it is not clear whether aging influences the effect of these treatments. There is evidence that multimodal rehabilitation, including classic massage, transcutaneous electrical nerve stimulation, and ultrasound therapy improves pain and function in older women (aged >60) suffering from back pain (Cichon et al., 2019). A systematic review found limited evidence of pain-reducing effects of physical therapy (three studies, two of them applying soft tissue treatments) among older adults with dementia (Coronado et al., 2019). In summary, we can only hypothesize that the fascial tissue of elderly people is susceptible to soft tissue stimuli, but its effect has to be determined.

## Conclusions

Aging is associated with metabolic, structural, and functional modifications of cells, tissues, and organs, which lead to a gradual decline in psycho-physical performance. In particular, the locomotory apparatus loses its effectiveness, due to the molecular and cellular changes occurring in the myofascia, the skeletal muscle tissue, the nervous system, and their structural and functional coupling. Genetics, epigenetics, environment, diseases, lifestyle, nutrition, and injuries also have a prominent role in tissue remodeling occurring with aging (Jee and Kim, 2017). Thanks to recent scientific progress, many phenomena and mechanisms associated with aging have been defined, but still much remains to be investigated. From the perspective of healthy aging, it is crucial to identify and hinder all the age-dependent modifications through specific strategies targeting etiologic factors, and also psycho-social issues. Indeed, ultrasound-based techniques can provide a detailed morphological characterization of skeletal muscle and connective tissue, thus allowing a specific analysis of the detrimental changes occurring in the myofascia with aging. Moreover, tissue manipulation techniques might contribute to improving myofascial regeneration in the elderly. Physical activity has been also suggested as an effective strategy for counteracting the deleterious consequences of aging, given that skeletal muscle plasticity might be only partially lost in elderly individuals (Distefano and Goodpaster, 2018). However, this issue is still debated, due to contradictory results. Nevertheless, the constant progress in technology and biomedical research holds great promise for fighting the burden of aging by targeted therapeutic interventions.

## Author Contributions

All authors contributed substantially to the design and concept of the review. WK, AZ, RS, and KH drafted the manuscript. SW and JF critically revised the manuscript. All authors approved the content of the final version of the manuscript. All authors agree to be accountable for all aspects for the work and ensure that all questions related to the accuracy or integrity of any part of the work are appropriately investigated and resolved.

## Conflict of Interest

The authors declare that the research was conducted in the absence of any commercial or financial relationships that could be construed as a potential conflict of interest.
